# Sheep as a large animal model for cochlear implantation

**DOI:** 10.1016/j.bjorl.2021.02.014

**Published:** 2021-03-26

**Authors:** Thuy-Tran Trinh, C. Cohen, L. Boullaud, J.P. Cottier, David Bakhos

**Affiliations:** a CHRU de Tours, Service d’ORL et Chirurgie Cervico-Faciale, France; b CHRU de Tours, Service de Neuroradiologie, France; c Université François Rabelais de Tours, France; d Inserm U1253, Tours, France

**Keywords:** Cochlear implant, Surgical training, Sheep, Animal model

## Abstract

**Introduction:**

In surgical training, large animal models are more suitable as their anatomy is more similar to humans. In otology, there have been relatively few studies about large animal models for surgical training.

**Objective:**

In this study, we aimed to do a neuroradiologic evaluation and surgical insertion of a cochlear implant electrode array on a sheep head model.

**Methods:**

Twenty cadaveric sheep heads were studied. A computed tomography scan and neuroradiologic evaluation was performed on each head, obtaining measurements of the inner ear for each sheep. Sheep measurements were compared to those from temporal bone computed tomography scans from 20 female humans. Surgical procedures were first trained with 13 of the sheep temporal bones, after which cochlear implantation was performed on the remaining 7 temporal bones. The position of the inserted electrode array insertion was confirmed by computed tomography scan after the procedure.

**Results:**

Neuroradiologic evaluation showed that, relative to the 20 female humans, the mean ratio for sheep was 0.60 for volume of cochlea, 0.70 for height of cochlea, 0.73 for length of cochlea; ratios for other metrics were >0.80. For the surgical training, the round window was found in all 20 sheep temporal bones. Computed tomography scans confirmed that electrode insertions were fully complete; the mean value of electrode array insertion was 18.3 mm.

**Conclusion:**

The neuroradiologic and surgical training data suggest that the sheep is a realistic animal model to train cochlear implant surgery and collection of perilymph samples, but less so for surgical training of mastoidectomy due to pneumatization of the mastoid.

## Introduction

The sheep is already known worldwide as an animal model for research studies, such as the famous cloned sheep “Dolly”.^1^ While sheep are used in many studies, murine species is the more common animal model in laboratory studies. However, murine species exhibit only a few anatomical, biological, and metabolic similarities to humans. In otology, the most common animal models are guinea pig and Mongolian gerbil.^2^, ^3^ The easy access to the inner ear make them suitable animal models despite their higher frequency hearing range.^4^ However, the dimensions of their inner ear are smaller than in humans, makingit more difficult to conduct with biomedical devices such as the cochlear implant (CI). While large animals can be disadvantageous in terms of housing or managing, they are more similar to humans in terms of anatomic dimensions and physiology.^5^ Large animals like sheep may also be appropriate to be a model of hearing loss given the anatomical and physiological similarities to the human cochlea. Cordero et al.^6^ described sheep ears as a potentially good animal model for stapedectomy training due to relatively low overall costs, easy access to the stapes, and anatomic similarities to human. Indeed, the sheep presents similar inner and middle ear anatomy as found in humans, with all inner ear structures identified.^7^, ^8^, ^9^ Physiologically, the hearing frequency spectrum is also comparable between sheep and humans.^10^ The frequency spectrum for auditory brainstem responses (ABRs) have also been shown to be similar between sheep and humans;^11^ however, wave peak latencies and amplitudes vary between sheep and humans. Griffith et al.^11^ recorded ABRs in developing sheep. During the first seven weeks of life, wave peak amplitudes increased, and latencies decreased, indicating a maturation of the central auditory pathways. However, unlike in humans, wave V was absent in sheep.

In otology, relatively few surgical and/or research studies have been conducted with large animal models. The mastoid approach has been compared between pig and sheep, with sheep favored as a model for otologic surgery.^12^, ^13^ The mastoid approach is more difficult in pigs due to the abundance of fat tissue and the lack of mastoid pneumatization. In sheep, the mastoid is also poorly pneumatized, making middle ear access more difficult. However, the middle ear is quite similar to that of humans: the ossicular chain is well- defined and the round window (RW) can be accessed via the middle ear. The RW is easily identified once in the hypotympanum, allowing for sampling of perilymph fluid. The sheep inner ear is also similar to humans.^7^ As such, sheep would seem to be a good model for extraction of perilymph fluid (for metabolic analysis) and/or training for cochlear implantation and insertion of the electrode array.

Only two previous studies have performed inner ear computed tomography (CT) scan analysis in large animals: sheep in Seibel et al.,^7^ and macaque monkeys in Marx et al.^14^ Inner ear anatomical dimensions were smaller than in humans, but sufficient to allow for electrode insertion of a cochlear implant (CI), as in humans. In this study, we propose sheep as a large animal model for cochlear implantation and electrode insertion. We performed neuroradiological analysis using CT scans of temporal bones (TBs) to better understand inner ear anatomy of sheep, relative to humans. We successfully performed cochlear implantation and electrode insertion in a subset of the sheep TBs.

## Methods

Ten sheep cadaveric heads (20 TBs in total) were obtained from the Experimental Unity of Animal Physiology of Orfrasiere. A CT-scan was performed on each head, then TB drilling was performed on the first 13 TBs and the last 7 TBs were surgically implanted, a postoperative CT scan was then performed on those 7 implanted TBs.

Each procedure is described below.

### Computed Tomography (CT) scan procedure

**Figure 1:**
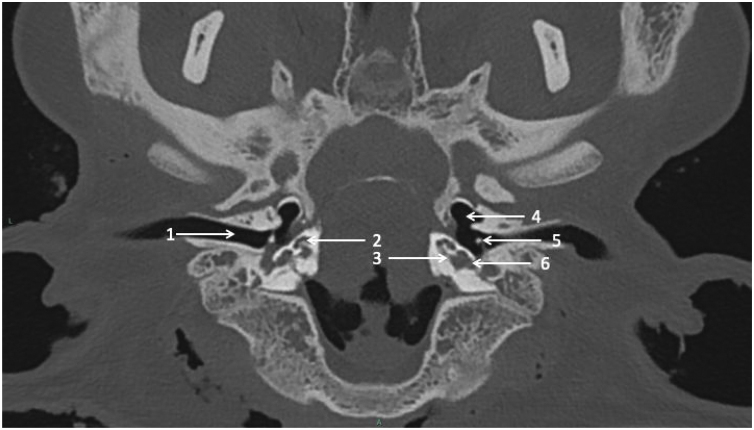
Cadaveric head axial sheep CT scan centered on cochleae.1 = external ear canal; 2 = right cochlea; 3 = basal turn of left cochlea; 4 = hypotympanum; 5 = malleus on tympanic membrane; 6 = round window membrane.

Cadaveric sheep heads were maintained on a tray as in a dorsal decubitus position. CT scans were performed on CIRE platform in INRA center of XXX using a Siemens Somatom® CT. The acquisition protocol was as follows: tube voltage = 140 kV, tube current = 400 mAs/slice, pitch = 0.35, rotation time = 1 s, field of view = 300 mm, window = 24.7 × 10.7 cm, matrix = 512 × 512, section thickness = 0.6 mm, increment = 0.1 mm. CT scans were performed before (Fig. 1) and after cochlear implantation.

The sheep radiological inner ear anatomy was compared to human anatomy. Existing TB CT scans were selected from our database of CI patients with the same acquisition protocol as above; the CT scans were performed as part of pre-implant assessment. Only female CT scans were selected to control for anatomic differences across human males and females’ sex, and to be consistent with the female sheep.

### Neuroradiologic evaluation

**Figure 2:**
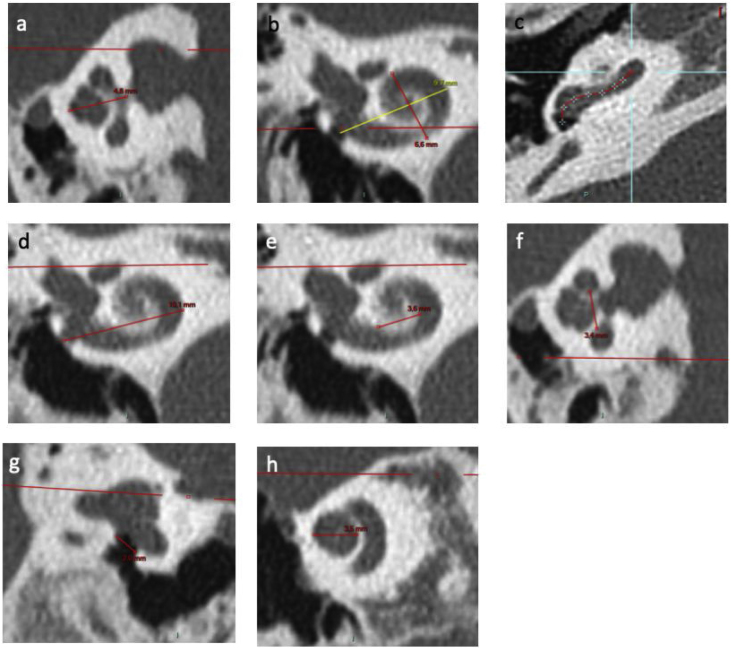
Inner ear CT scan measurements. (a) Height of cochlea on an axial view; (b) Measurements of length A on a reconstruction through modiolus. B measurement corresponds to width of cochlea; (c) Identification of points in basal turn of cochlea via the application “vessel analysis” to measure length of unrolled cochlea; (d) External diameter of basal turn of cochlea on a frontal view; (e) Internal diameter of basal turn of cochlea on a frontal view; (f) Internal diameter of basal turn of cochlea on a sagittal view; (g) RW diameter on a reconstruction from Saylisoy et al.^16^; (h) Diameter of apical turn of cochlea.
2) the width of cochlea (Fig. 2b) was quantified perpendicular to “A”, defined as the longest measurement between the round window (RW) and the lateral wall of cochlea through the modiolus,
3) the length of cochlea (Fig. 2c) was quantified by “unrolling” the cochlea using the “vessels analysis” tool in the PACS MIRC Carestream software and measuring the mean value between the central and external lumen. The length of cochlea on 720° was calculated according to previous studies^15^ (length = 3.65*A - 3.63),
4) the diameter of external basal turn (Fig. 2d) was quantified in terms of the mean value between the axial and sagittal plane of the longest measurement between the RW and the lateral wall of basal turn,
5) the diameter of the internal basal turn was quantified in terms of the mean value between the frontal (Fig. 2e) and sagittal planes (Fig. 2f) of the longest measurement between medial wall of basal turn.
6) the diameter of the RW (Fig. 2g) was quantified according to the strict plane described by Saylisoy et al.^16^,
7) the diameter of apical turn (Fig. 2h) was quantified in terms of the distance between the lateral walls through the modiolus, on a plane through the apex,
8) the number of spiral turns was calculated from the RW to apex, with one turn counted each time the spiral passed through the RW plane, and
9) the volume of cochlea was quantified using 3D-Doctor (version 3.5; Able Software Corp., Lexington, MA, USA) after interactive segmentation of cochlea.

**Figure 3:**
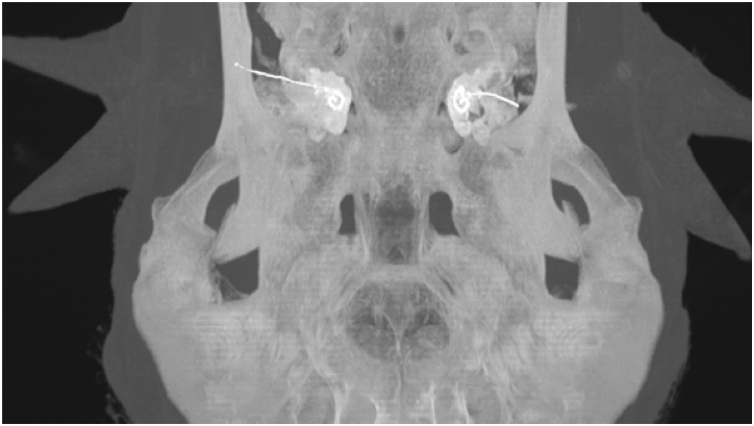
Sheep temporal bone CT scan with electrode arrays inserted in cochleae.

Computed tomography scans were evaluated by two experienced examiners using PACS MIRC Carestream (version 11.4.1.0324). For each sheep or human TB, several dimensions of the cochlea were quantified as follows:

1) the height of cochlea (Fig. 2a) was quantified as the mean distance between the axial and sagittal planes of the measurement perpendicular to the line outcropping the basal turn,

Electrode insertion was reviewed using postoperative CT scans (Fig. 3) and insertion depth was estimated using the “vessels analysis” tool in the PACS MIRC Carestream software.

Student’s *t*-tests were used to compare cochlea dimensions between sheep and humans. For each dimension, a ratio was calculated between the mean sheep and mean human values.

### Temporal bone drilling procedure

**Figure 4:**
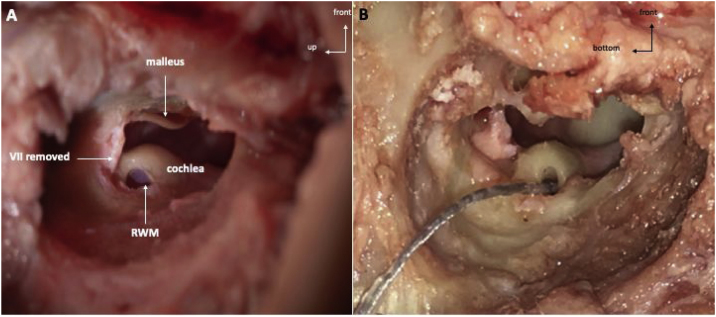
A, Intraoperative view of the RWM on a left sheep temporal bone. B, Electrode array completely inserted in the RWM on a left sheep cochlea. RWM, Round window membrane; EEC, External ear canal.

Following a posterior auricular incision, the surgical procedure was performed similarly to human approach. Mastoidectomy was performed using an otologic hand drill (Medtronic® Xomed) with a 6 mm cutting burr, following the external ear canal to discover hypotympanum bulla. The ossicular chain was exposed by drilling towards the top using a 3 mm ultra-diamond burr, and the RW was exposed by drilling backward. The RW visualization was optimized by removal of the facial nerve (FN) and labyrinth block (Fig. 4A).

The RW membrane was incised and the electrode array was inserted (AB HF MS Demo Electrode Assembly, manufactured by Advanced Bionics, California, USA) (Fig. 4B). The electrode array was a human demo device composed of 16 electrodes; the total length was 23.7 mm, and the active length was approximately 15 mm. We noted mastoid pneumatization, the ossicular chain, FN canal and RW were visible. We also noted if electrode insertion was complete.

## Results

### Computed tomography scans

Computed tomography scans from 20 sheep and 20 female TBs were analyzed; of the 20 sheep TBs, 7 were implanted. Inner ear morphologic study showed the presence of the 3 semi-circular canals, the vestibule, and the cochlea with 2.5 turns for all TBs. Measurement values were averaged across the two examiners and are shown for sheep in Table 1 and for human in Table 2. Student’s *t*-tests were used to compare measurements between human and sheep. All measurements were significantly larger for human TBs (*p* < 0.001), except for RW diameter (dF = 38, *t* = 1.51, *p* = 0.14). Mean data from sheep and human TBs were compared in terms of ratios. For sheep, the mean cochlea volume was 0.59, the mean height was 0.69, and the mean length was 0.73, relative to human; for the remaining dimensions, sheep ratios ranged from 0.81 to 1.1, relative to human.

**Table 1:** Sheep inner ear radiological measurements on temporal bone CT-scan.

Sheep TB	Age (year)	Side	Number of spiral turn	Height (mm)	Length measured (mm)	Length A (mm)	Calculated length Jolly (mm)	Width (mm)	External diameter of basal turn (mm)	Internal diameter of basal turn (mm)	Diameter of apical turn (mm)	RW diameter (mm)	Volume (mm^3)^
1	2	R	2.5	2.06	20.42	7.77	24.71	5.08	6.93	2.79	2.78	2.14	63
2	2	L	2.5	2.43	19.37	7.71	24.51	5.04	6.56	2.3	2.97	1.98	62
3	2.75	R	2.5	2.15	21.51	7.83	24.95	5.59	6.48	2,44	2,78	1,89	73
4	2.75	L	2.5	2.65	20.95	7.92	25.28	5.44	6.98	2.41	2,91	1,77	73
5	2.75	R	2.5	2.47	19.92	7.61	24.15	5.24	6.65	2.44	2,75	1,96	44
6	2.75	L	2.5	2.45	20.26	7.78	24.77	5.29	6.86	2.37	2,93	1,97	43
7	2.75	R	2.5	2.52	21.13	7.72	24.55	5.13	7.25	2.38	2,98	1,76	47
8	2.75	L	2.5	2.4	19.66	7.48	23.67	5.53	7.31	2.37	2,92	1,72	54
9	2.75	R	2.5	2.47	21.79	7.22	22.7	5.1	6.68	2.32	2,74	1,77	40
10	2.75	L	2.5	2.49	19.69	7.22	22.7	5.18	6.71	2.58	2,74	1,58	37
11	2.25	R	2.5	2.37	20.99	7.67	24.37	4.78	7.19	2.57	2,78	1,69	44
12	2.25	L	2.5	2.51	19.45	7.4	23.36	4.94	7.26	2.43	2,92	1,55	42
13	4	R	2.5	2.36	19.01	6.8	21.19	5.02	6.8	2.31	2,61	2,28	47
14	4	L	2.5	2.53	20.92	7.43	23.47	4.83	7.12	2.75	2,91	2,05	45
15	4	R	2.5	2.1	21.4	8.3	26.67	5.14	7.25	2.73	2,92	1,73	46
16	4	L	2.5	2.27	21.58	8.34	26.79	5.13	7.34	2.69	2,9	1,7	44
17	5	R	2.5	2.6	22.37	7.84	24.97	5.01	7.4	2.43	2,75	1,8	37
18	5	L	2.5	2.45	22.46	8.22	26.35	5.5	7.48	2.83	2,83	1,78	38
19	6	R	2.5	2.83	21.09	7.45	23.54	4.89	7.91	2.87	2,89	1,78	43
20	6	L	2.5	2.7	20.76	7.74	24.6	5.69	7.65	2.48	2,83	1,69	40
Mean	3.425		2.5	2.44	20.74	7.67	24.37	5.18	7.11	2.52	2,84	1,83	48,1
SD	1.26		0	0.19	0.97	0.37	1.34	0.25	0.39	0.18	0,095	0,18	10,8

TB, Temporal bone; R, Right; L, Left; mm, millimeter; RW, Round window; SD, Standard deviation.

**Table 2:** Woman inner ear radiological measurements on temporal bone CT-scan.

Human TB	Age (year)	Side	Number of spiral turn	Height (mm)	Length measured (mm)	Length A (mm)	Calculated length Jolly (mm)	Width (mm)	External diameter of basal turn (mm)	Internal diameter of basal turn (mm)	Diameter of apical turn (mm)	RW diameter (mm)	Volume (mm^3)^
1	32	R	2.5	3.64	28.48	9.87	32.38	7.28	7.92	3.2	3,5	1,72	74
2	32	L	2.5	3.48	29.79	9.9	32.49	7.22	8.22	3.13	3,58	1,65	95
3	40	R	2.5	3.36	26.67	8.8	28.47	6.54	7.45	2.91	3,54	1,59	78
4	40	L	2.5	3.41	26.81	8.93	28.96	6.68	7.57	2.75	3,55	1,68	71
5	52	R	2.5	3.63	28.33	9.07	29.46	6.56	7.73	2.94	3,62	1,7	57
6	52	L	2.5	3.69	27	8.99	29.17	6.52	7.37	3.08	3,37	1,65	73
7	36	R	2.5	3.57	29.47	9.66	31.63	6.87	7.78	2.61	3,62	1,65	94
8	36	L	2.5	2.84	29.41	9.35	30.48	6.56	7.83	2.58	3,52	1,72	85
9	38	R	2.5	3.28	28.12	9.49	31.01	6.33	7.02	3.12	3,36	1,97	78
10	38	L	2.5	3.11	27.74	9.32	30.39	6.31	7.57	2.94	3,52	1,87	84
11	21	R	2.5	3.46	27.19	9.44	30.81	6.49	7.87	2.64	3,66	1,7	74
12	21	L	2.5	3.39	28.98	8.97	29.11	6.63	7.7	2.57	3,67	1,67	64
13	65	R	2.5	3.79	29.4	9.14	29.73	6.75	7.95	2.64	3,63	2,06	94
14	65	L	2.5	3.7	29.6	9.36	30.52	6.84	7.7	2.57	3,58	2,33	88
15	38	R	2.5	3.39	27.56	9.28	30.22	6.25	7.43	2.54	3,4	1,82	90
16	38	L	2.5	3.65	27.22	9.1	29.57	6.61	7.39	2.61	3,35	1,92	92
17	83	R	2.5	3.39	27.37	9.25	30.13	6.6	7.41	2.59	3,4	1,72	83
18	83	L	2.5	3.36	29.51	9.61	31.45	6.27	7.05	2.53	3,45	1,19	97
19	31	R	2.5	3.71	28.46	9.06	29.42	6.16	6.99	2.64	3,3	1,49	91
20	31	L	2.5	3.53	27.54	8.98	29.15	6.19	7.11	2.55	3,43	1,47	71
Moyenne	43.6		2.5	3.47	28.23	9.28	30.23	6.58	7.55	2.76	3,50	1,73	81,65
DS	17.82		0	0.23	1.05	0.31	1.14	0.31	0.34	0.23	0,11	0,24	11,2

TB, Temporal bone; R, Right; L, Left; mm, millimeter; RW, Round window; SD, Standard deviation.

### Sheep cadaveric temporal bone drilling

Drilling was performed in 20 sheep TBs (10 right, 10 left), and electrode arrays were inserted in 7 of the 20 sheep TBs; the first 13 TBs were used to train surgical procedures. In the first surgical procedure, the external ear canal posterior wall was drilled, leading to difficulty in finding the RW; to find the RW membrane, all structures were then drilled. In the second procedure, the external ear canal was identified and the ossicular chain was revealed. For the 7 TBs that were implanted, electrode insertion was visually complete. The length of the inserted electrode array was measured; the mean value was 18.3 ± 1.5 mm. No mastoid pneumatization was observed in any of the drilled sheep TBs. The RW was found in all procedures and the FN canal was found in 80% of procedures. All electrodes (16/16) were successfully inserted in the 7 cochlear implantations. The characteristics of the surgical procedures are shown in Table 3.

**Table 3:** Anatomical structures identified during the temporal bone drilling.

Sheep TB	Age (year)	Side	Mastoid pneumatisation	Ossicular chain	FN canal	RW visibility	Easyness of electrode insertion	Electrode insertion (n = electrode inserted)	Length of electrode array inserted (mm)
1	2	R	–	–	–	++	/	/	/
2	2	L	–	–	+	++	/	/	/
3	2.75	R	–	–	+	++	/	/	/
4	2.75	L	–	–	+	++	/	/	/
5	2.75	R	–	–	++	++	/	/	/
6	2.75	L	–	–	++	+	/	/	/
7	2.75	R	–	–	+	++	/	/	/
8	2.75	L	–	–	–	+	/	/	/
9	2.75	R	–	–	++	++	/	/	/
10	2.75	L	–	–	++	+	/	/	/
11	2.25	R	–	–	++	+	/	/	/
12	2.25	L	–	–	++	++	/	/	/
13	4	R	–	++	++	++	/	/	/
14	4	L	–	+	++	++	Y	Complete (n = 16)	19.6
15	4	R	–	++	++	++	Y	Complete (n = 16)	18.6
16	4	L	–	++	–	++	Y	Complete (n = 16)	19
17	5	R	–	++	++	++	Y	Complete (n = 16)	18.8
18	5	L	–	–	–	+	Y	Complete (n = 16)	19.3
19	6	R	–	++	++	++	Y	Complete (n = 16)	17
20	6	L	–	–	+	++	Y	Complete (n = 16)	15.5

TB, Temporal Bone; R, Right; L, Left; (-), not seen; (+), partial visibility; (++), complete visibility; mm, millimeter; Y, Yes. Grey underlying: sheep TBs with cochlear implantation.

## Discussion

Cochlea dimensions were consistently smaller in sheep than in human, but generally comparable. Other dimensions were comparable to those described in previous studies, as shown in Table 4.

**Table 4:** Dimensions of cochlea in other mammalian species.

	Width ± SD (mm)	Height ± SD (mm)	Length ± SD (mm)	Turns
Guinea pig	#	#	#	3 ¼ ^17^
				3 ½ – 3 ¾^18^
Mongolian Gerbil	2.6 ± 0.1^19^	3.4 ± 0.1^19^	10.9 ± 0.43^19^	3 ¼ ^19^
			11.1 ± 0.54^4^	2 ½ – 3 ^20^
Cat	#	4.45 ± 0.24 ^21^	23 ± 2.26^21^	3 ½ – 3 ¾ ^21^
Human	7.0 ± 0.3 ^22^	3.8 ± 0.2 ^22^	35.6 ± 1.4^23^	2 ½ – 3 ^23^
Human (our study)	6.58 ± 0.31	3.47 ± 0.23	30.2 ± 1.14	2 ½
Sheep	#	#	19.9 ± 1.7^7^	2 ½ ^9^
Sheep (our study)	5.18 ± 0.25	2.44 ± 0.19	20.74 ± 0.97	2 ½

The surgical procedure progressively improved with successive drillings. The ossicular chain was not visible during drilling, as the RW was in a posterior orientation in the hypotympanum. The ossicular chain, particularly the malleus, is more visible when the RW orientation is more anterior, because of anatomical variations. The RW was well observed, suggesting that surgical training for electrode array insertion or extraction of perilymph samples could be performed in sheep. However, the sheep appears to be an unsuitable animal model for mastoidectomy as the mastoid is poorly pneumatized.^13^

The mean ratio between sheep and human cochlea volume was 0.59, consistent with previous research.^7^ This relatively small ratio (compared to the other dimensions) may be because CT scan is not the gold standard for evaluating inner ear fluid. MRI using hyper-weighted T2 sequences would be more appropriate and may reveal more similar endocochlear fluid volumes between sheep and human.

The sheep appears to be a good animal model for inner ear experimentation and surgical training, especially for CI electrode insertion. Indeed, the length of the “unrolled” cochlea for sheep is closer to that in human than in other small animals (Table 4). Given the comparable hearing spectrum between sheep and human, tonotopy should also be comparable. Moreover, because the cochlea dimensions approach those of human, the same implant device used in humans can be used in sheep, which is useful for training CI electrode insertion. In cat, the length of the cochlea is closer to that of human, but the number of spiral turns is higher, which could lead to more traumatic in electrode insertion. In addition, cat models are generally less accessible due to ethical reasons.

Given the comparable cochlear dimensions between sheep and human, sheep are useful for obtaining a sufficient volume of perilymph fluid for analysis. Moreover, there was no significant difference in RW dimension between sheep and human (*p* = 0.14). and the RW was found in each drilling procedure, making sheep a good model for any surgery involving the RW (e.g., cochlear implantation, sampling of perilymph fluid, etc.).

Using a human cochlear implant device, all 16 electrodes were visually inserted in sheep TBs, with an average insertion depth of 18.3 mm. In the guinea pig, cochlear implantation been has performed with a device that included only 2 active electrodes, for an electrode array length of 4 mm^2^. DeMason et al.^3^ implanted gerbils with 1–2 platinum iridium contacts in order to observe cochlear trauma during insertion and to evaluate the functional status of the electrodes; the deepest insertions electrodes were approximately 4.1 mm. Insertion depth and cochlea trauma are particularly interesting in the context of preservation of residual acoustic hearing. With the sheep model, a human device can be used, allowing for insights regarding insertion depth and trauma relevant to human CI recipients.

The effects of hearing loss have been studied in many animal models. To better understand the physiopathology, hearing loss has been induced by aminoglycosides in guinea pig^17^ or noise-induced in mice,^18^ or vice versa. Using sheep as an animal model of hearing loss would be interesting. Indeed, as sheep appear to have comparable inner ear anatomy and auditory physiology to humans, sheep may be a valuable animal model to study sensorineural hearing loss, particularly the injury site, which is difficult to measure in post-lingual adults. The next step would be to introduce a CI for in vivo model of hearing loss using sheep.^19^ Developing a sheep model may be also useful for pharmacometabolomics studies of hearing loss. Information from such studies could be useful in develop therapeutic drug delivery in situ using, according to etiology of deafness; corticosteroids could be delivered via the implanted electrodes to reduce cochlear trauma when there is residual acoustic hearing.^20^

While sheep may be a promising animal model, their size can be challenging in terms of maintenance. Sheep require a fold for housing and an experienced staff. Moreover, using sheep in research is more restricted than using mice because of the cost and ethical considerations. Large animals have been previously studied in otology. Although using sheep for *in vivo* experiences is a real challenge, some authors studied utriculostomy on sheep and kept sheep alive 3 months after surgical procedure in order to evaluate nystagmus, general state and balance.^21^ Angeli et al.^22^ studied electrocochleography while Maia & Lavinsky studies distortion product otoacoustic emissions^23^, ^24^ during induced hyperinsulinism on anesthetized sheep and concluded that hyperinsulinism suppressed cochlear function. Gurr et al.^12^ studied the anatomy of pig TBs in a surgical context. The middle ear is very similar to human, especially the ossicular chain, where the malleus, incus and stapes have the same morphology as in humans. The FN canal can be observed in front of the RW, as in human. However, similar to our finding in sheep, the pig mastoid is even less pneumatized, the classical landmarks could not be found, and it was necessary to remove the atlanto-occipital joint. The present data suggest that sheep may be a better large animal model than pig. Further studies are needed to further validate sheep as a realistic animal model.

## Conclusion

Sheep appear to be a good animal model for training cochlear implantation. The visibility and accessibility of the RW are easy and could permit sampling of perilymph fluid. The similarity to human inner ear anatomy and physiology suggest that sheep could also be a good model and deliver therapeutic drugs to the inner ear via CI.

## Conflicts of interest

The authors declare no conflicts of interest.
